# ﻿A new species of the *Cyrtodactyluschauquangensis* species group (Squamata, Gekkonidae) from Lao Cai Province, Vietnam

**DOI:** 10.3897/zookeys.1192.117135

**Published:** 2024-02-19

**Authors:** Tung Thanh Tran, Quyen Hanh Do, Cuong The Pham, Tien Quang Phan, Hanh Thi Ngo, Minh Duc Le, Thomas Ziegler, Truong Quang Nguyen

**Affiliations:** 1 Vinh Phuc College, Phuc Yen City, Vinh Phuc Province, Vietnam; 2 Institute of Ecology and Biological Resources, Vietnam Academy of Science and Technology, 18 Hoang Quoc Viet Road, Hanoi, Vietnam; 3 Graduate University of Science and Technology, Vietnam Academy of Science and Technology, 18 Hoang Quoc Viet Road, Cau Giay, Hanoi, Vietnam; 4 Central Institute for Natural Resources and Environmental Studies, Vietnam National University, 19 Le Thanh Tong, Hanoi, Vietnam; 5 Cologne Zoo, Riehler Straße 173, 50735, Cologne, Germany; 6 Institute of Zoology, University of Cologne, Zülpicher Straße 47b, 50674, Cologne, Germany; 7 Faculty of Environmental Sciences, Hanoi University of Science, Vietnam National University, 334 Nguyen Trai Road, Hanoi, Vietnam; 8 Department of Herpetology, American Museum of Natural History, Central Park West at 79th Street, New York, New York 10024, USA

**Keywords:** *Cyrtodactylusluci* sp. nov., gecko, molecular phylogeny, morphology, ND2 gene, taxonomy

## Abstract

We describe a new species of the genus *Cyrtodactylus* based on five adult specimens from Bac Ha District, Lao Cai Province, northern Vietnam. *Cyrtodactylusluci***sp. nov.** is distinguished from the remaining Indochinese bent-toed geckos by a combination of the following morphological characteristics: medium size (SVL up to 89.5 mm); dorsal tubercles in 17–19 irregular transverse rows; ventral scales in 32–34 longitudinal rows at midbody; precloacal pores present in both sexes, 9 or 10 in males, 8 or 9 in females; 12–15 enlarged femoral scales on each thigh; femoral pores 9–12 in males, 5–10 in females; postcloacal tubercles 2–4; lamellae under toe IV 21–23; dorsal pattern consisting of 5 or 6 irregular dark bands, a thin neckband without V-shape or triangle shape in the middle, top of head with dark brown blotches; subcaudal scales transversely enlarged. Molecular phylogenetic analyses recovered the new species as the sister taxon to *C.gulinqingensis* from Yunnan Province, China, with strong support from all analyses and the two taxa are separated by approximately 8.87–9.22% genetic divergence based on a fragment of the mitochondrial ND2 gene. This is the first representative of *Cyrtodactylus* known from Lao Cai Province.

## ﻿Introduction

The *Cyrtodactyluschauquangensis* species group is broadly distributed in the northern Indochina-Burma region, from northern Thailand and Laos to north central and northwestern Vietnam and to southwestern China (Uetz et al. 2023). Taxa within the group are almost exclusively adapted to karst ecosystems. Le et al. (2016) suggested that the group included at least ten species. Grismer et al. (2021a, 2021b) provided a taxonomic review and analyzed phylogenetic relationships of 17 species and one undescribed form from northern Thailand. The group currently contains 23 recognized species with several taxa recently discovered from Yunnan Province, southern China (Grismer et al. 2021a, 2021b, 2021c; Liu and Rao 2021, 2022).

Lao Cai Province is located in the border area between Vietnam and China with an international borderline of 203 km (Portal of Lao Cai Province 2023). Although Lao Cai contains an area of limestone forest (Portal of Lao Cai Province 2023), no representative of *Cyrtodactylus* has been known from this province so far. On the other hand, members of the genus have been recorded in several neighboring forests, including six species from Yunnan Province of China (*Cyrtodactylusdianxiensis* Liu & Rao, 2021, *C.gulinqingensis* Liu, Li, Hou, Orlov & Ananjeva, 2021, *C.hekouensis* Zhang, Liu, Bernstein, Wang & Yuan, 2021, *C.menglianensis* Liu & Rao, 2022, *C.wayakonei* Nguyen, Kingsada, Rösler, Auer & Ziegler, 2010, *C.zhenkangensis* Liu & Rao, 2021) and five other species reported from Vietnam: one species from Lai Chau (*C.martini* Ngo, 2011) and four species from Son La (*C.bichnganae* Ngo & Grismer, 2010, *C.otai* Nguyen, Le, Pham, Ngo, Hoang, Pham & Ziegler, 2015, *C.sonlaensis* Nguyen, Pham, Ziegler, Ngo & Le, 2017 and *C.taybacensis* Pham, Le, Ngo, Ziegler & Nguyen, 2019).

During our recent field trip in northern Vietnam, we collected five specimens of an unnamed gekkonid species from Bac Ha District, Lao Cai Province, which can be assigned to the *Cyrtodactyluschauquangensis* group based on molecular data. However, the population from Lao Cai Province can be distinguished from congeners by morphological differences and genetic divergence. Therefore, we describe it as a new species in the following.

## ﻿Materials and methods

### ﻿Sampling

**Figure 1.**
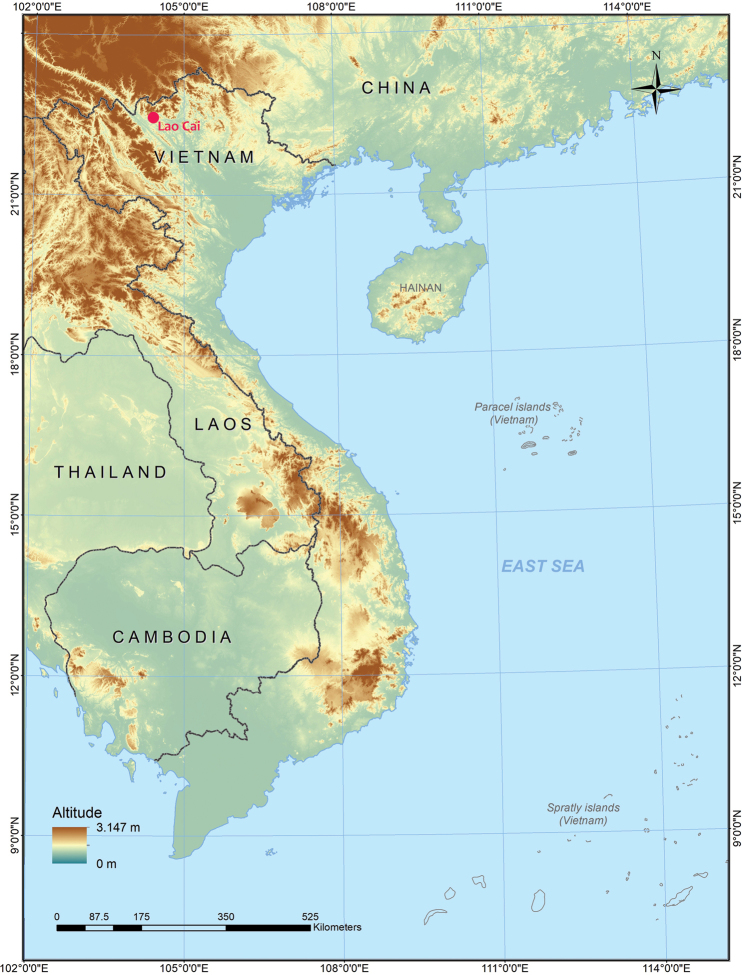
Type locality of *Cyrtodactylusluci* sp. nov. in Lao Cai Province (red circle), Vietnam.

Field surveys were conducted in Bac Ha District, Lao Cai Province, Vietnam in June 2022 and October 2023 (Fig. 1). After being photographed in life, specimens were anesthetized and euthanized in a closed vessel with a piece of cotton wool containing ethyl acetate (Simmons 2002), fixed in 85% ethanol and subsequently stored in 70% ethanol. Specimens were subsequently deposited in the collections of the Institute of Ecology and Biological Resources (**IEBR**), Hanoi, Vietnam.

### ﻿Molecular data and phylogenetic analyses

DNA was extracted using DNeasy Blood and Tissue kit (Qiagen, Germany) following manufacturer’s instructions. Extracted DNA was amplified by HotStar Taq Mastermix (Qiagen, Germany) with 21 µl volume (10 µl of mastermix, 5 µl of water, 2 µl of each primer at 10 pmol and 2 µl of DNA). PCR conditions were: 95 °C for 15 min to active the taq; with 40 cycles at 95 °C for 30 s, 52 °C for 45 s, 72 °C for 60 s; and the final extension at 72 °C for 6 min. A fragment of the mitochondrial gene, NADH dehydrogenase subunit 2 (ND2), was amplified using the primer pair MetF1 (5’-AAGCTTTCGGGCCCATACC-3’) and COIR1 (5’-AGRGTGCCAATGTCTTTGTGRTT-3’) (Arevalo et al. 1994; Macey et al. 1997). PCR products were visualized using electrophoresis through a 2% agarose gel stained with ethidium bromide. Successful amplifications were purified to eliminate PCR components using GeneJET^TM^ PCR Purification kit (ThermoFischer Scientific, Lithuania). Purified PCR products were sent to FirstBase (Malaysia) for sequencing in both directions. We included two samples of the newly discovered population from Lao Cai Province, one of *Cyrtodactylusbichnganae*, one of *C.bobrovi*, one of *C.cucphuongensis*, one of *C.huongsonensis*, one of *C.ngoiensis*, one of *C.sonlaensis*, one of *C.taybacensis*, and one of *C.vilaphongi* along with all available GenBank sequences of these species and other members of the *Cyrtodactyluschauquangensis* group. Two species, *C.hontreensis* and *C.septimontium*, of the *C.intermedius* group, were selected as outgroups (Grismer et al. 2021b). In the end, we were able to incorporate all ingroup taxa (Table 1).

**Table 1.** Species of Cyrtodactylus used in the phylogenetic analysis including localities and GenBank accession numbers of the mitochondrial NADH dehydrogenase subunit 2 (ND2) fragment gene (–: data unavailable).

Species	Locality	Museum number/Field number	Accession number	Reference
*C.auribalteatus*	Cambodia: Phnom Aural Wildlife Sanctuary, Kampong Speu Province	–	AP018116	Areesirisuk et al. 2018
*Cyrtodactylusluci* sp. nov.	Vietnam: Coc Ly Commune, Bac Ha District, Lao Cai Province	IEBR R.5240	PP253960	This study
*Cyrtodactylusluci* sp. nov.	Vietnam: Coc Ly Commune, Bac Ha District, Lao Cai Province	IEBR R.5241	PP253059	This study
*C.bichnganae*	Vietnam: Son La City, Son La Province	UNS 0473	MF169953	Brennan et al. 2017
*C.bichnganae*	Vietnam: Son La City, Son La Province	TBU PAT250	PP253951	This study
*C.bobrovi*	Vietnam: Ngoc Son – Ngo Luong NR, Lac Son District, Hoa Binh Province	IEBR A.2015.29	MT953471	Grismer et al. 2020
*C.bobrovi*	Vietnam: Tan Lac, Hoa Binh Province	HB.2015.73	PP253953	This study
*C.chauquangensis*	Vietnam: Quy Hop District, Nghe An Province	NA 2016.1	MT953475	Grismer et al. 2020
*C.cucphuongensis*	Vietnam: Cuc Phuong NP, Ninh Binh Province	CP 17.02	MT953477	Grismer et al. 2020
*C.cucphuongensis*	Vietnam: Cuc Phuong NP, Ninh Binh Province	NHQ.17.71	PP253954	This study
*C.doisuthep*	Thailand: Doi Phrabart abbey, Chiang Dao District, Chiang Mai Province	AUP–00777	MT497801	Chomdej et al. 2021
*C.doisuthep*	Thailand: Doi Suthep Mt., Chiang Mai Province	AUP–00774	MT550626	Chomdej et al. 2020
*C.dumnuii*	Thailand: Chiang Dao, Chiang Mai Province	AUP 00768	MW713972	Grismer et al. 2021
*C.erythrops*	Thailand: Coral Cave, Pang Mapha District, Mae Hong Son Province	AUP–00771	MT497806	Chomdej et al. 2021
*C.erythrops*	Thailand: Moe Cham Pae, Mae Hong Son	AUP 00772	MW713958	Grismer et al. 2021b
*C.gulinqingensis*	China: Gulinqing NR, Maguan County, Wenshan Prefecture, Yunnan Province	KIZ 061813	MZ782150	Liu et al. 2021
*C.gulinqingensis*	China: Gulinqing NR, Maguan County, Wenshan Prefecture, Yunnan Province	KIZ 061816	MZ782152	Liu et al. 2021
*C.gulinqingensis*	China: Gulinqing NR, Maguan County, Wenshan Prefecture, Yunnan Province	KIZ 061817	MZ782153	Liu et al. 2021
*C.houaphanensis*	Laos: near Viengxai, Houaphan Province	IEBR A.2013.109	MW792067	Grismer et al. 2021b
*C.huongsonensis*	Vietnam: Huong Son, My Duc District, Hanoi City	IEBR A.2011.3A	MT953481	Grismer et al. 2020
*C.huongsonensis*	Vietnam: Lac Thuy, Hoa Binh Province	HB.2016.44	PP253957	This study
*C.hontreensis*	Vietnam: Hon Tre Island, Kien Hai District, Kien Giang Province	LSUHC8583	JX440539	Wood et al. 2012
*C.martini*	Vietnam: Lai Chau Town, Lai Chau Province	UNS 0471	MF169968	Brennan et al. 2017
*C.menglianensis*	China: Menglian County, Puer City, Yunnan Province	KIZ20210714	OM296043	Liu and Rao 2022
*C.menglianensis*	China: Menglian County, Puer City, Yunnan Province	KIZ20210716	OM296044	Liu and Rao 2022
*C.ngoiensis*	Laos: Ngoi District, Luang Prabang Province	IEBR A.20213.100	MW792066	Grismer et al. 2021b
*C.ngoiensis*	Laos: Ngoi District, Luang Prabang Province	AT2012.1	PP253956	This study
*C.otai*	Vietnam: Xuan Nha NR, Van Ho District, Son La Province	TBU 2017.2	MT953486	Grismer et al. 2020
*C.puhuensis*	Vietnam: Pu Hu Nature Reserve, Thanh Hoa Province	ND 01.15	MT953489	Grismer et al. 2020
*C.septimontium*	Vietnam: Co To Mountain, An Giang Province	NAP 05321	MH940237	Murdoch et al. 2019
*C.sonlaensis*	Vietnam: Muong Bang Commune, Phu Yen District, Son La Province	IEBR A.2017.1	MT953492	Grismer et al. 2020
*C.sonlaensis*	Vietnam: Muong Bang Commune, Phu Yen District, Son La Province	IEBR A.2017.2	PP253958	This study
*C.soni*	Vietnam: Van Long Wetland NR, Gia Vien District, Ninh Binh Province	IEBR R.2016.4	MT953491	Grismer et al. 2020
*C.spelaeus*	Laos: Kasi District, Vientiane Province	HLM 0315	MW713962	Grismer et al. 2021b
*C.taybacensis*	Vietnam: Ca Nang Commune, Quynh Nhai District, Son La Province	IEBR 4379	MT953495	Grismer et al. 2020
*C.taybacensis*	Vietnam: Ta Ma Commune, Tuan Giao District, Dien Bien Province	DB2021.1	PP253952	This study
*C.vilaphongi*	Laos: Luang Prabang District, Luang Prabang Province	NUOL R–2013.5	PP253955	This study
*C.vilaphongi*	Laos: Luang Prabang District, Luang Prabang Province	IEBR A.2013.13	MT953497	Grismer et al. 2021b
*C.wayakonei*	Laos: Ban Nam Eng, Vieng Phoukha District, Luang Nam Tha Province	ZFMK 91016	MT953498	Grismer et al. 2020
*C.zhenkangensis*	China: Zhenkang County, Lincang City, Yunnan Province	KIZL2020047	MW792062	Grismer et al. 2021b

After sequences were aligned by Clustal X v.2.1 (Thompson et al. 1997), data were analyzed using maximum likelihood (ML) as implemented in IQ-TREE (Nguyen et al. 2015), maximum parsimony (MP) implemented in PAUP*4.0b10 (Swofford 2001) and Bayesian inference (BI) as implemented in MrBayes v.3.2.7 (Ronquist et al. 2012). For the MP analysis, heuristic analysis was conducted with 100 random taxon addition replicates using tree-bisection and reconnection (TBR) branch-swapping algorithm, with no upper limit set for the maximum number of trees saved. Bootstrap support (BP) was calculated using 1000 pseudo-replicates and 100 random taxon addition replicates. All characters were equally weighted and unordered. For the ML analysis, we used IQ-TREE v.1.6.8 (Nguyen et al. 2015) with a single model and 10000 ultrafast bootstrap replications (UFB). The optimal model for nucleotide evolution was determined using jModelTest v.1.2.4 (Darriba et al. 2012).

For the BI analysis, we used the optimal model determined by jModelTest with parameters estimated by MrBayes v.3.2.7. Two independent analyses with four Markov chains (one cold and three heated) were run simultaneously for 10^7^ generations with a random starting tree and sampled every 1000 generations. Loglikelihood scores of sample points were plotted against generation time to detect stationarity of the Markov chains. Trees generated prior to stationarity were removed from the final analyses using the burn-in function. The posterior probability values (PP) for all nodes in the final majority rule consensus tree were provided. We regard BP ≥ 70% and UFB and PP of ≥ 95% as strong support and values of < 70% and < 95%, respectively, as weak support (Hillis and Bull 1993; Ronquist et al. 2012; Minh et al. 2013).

The optimal model for nucleotide evolution was set to GTR+I+G for ML and BI analysis. The cut-off point for the burn-in function was set to 60, or 0.6% of the total number of trees generated, in the Bayesian analysis, as -lnL scores reached stationarity after 60,000 generations in both runs. Uncorrected pairwise divergences were calculated in PAUP*4.0b10.

### ﻿Morphological characters

Measurements were taken with a digital calliper to the nearest 0.1 mm. Abbreviations are as follows: **SVL**: snout-vent length, measured from tip of snout to vent; **TaL**: tail length, measured from vent to tip of tail (* = regenerated); **HL**: head length, measured from tip of snout to retroarticular process of jaw; **HW**: head width, maximum width of head; **HH**: head height, from occiput to underside of jaws; **OrbD**: orbital diameter, greatest diameter of orbit; **SE**: snout to eye distance, from tip of snout to anterior-most point of eye; **EE**: eye to ear distance, from anterior edge of ear opening to posterior corner of eye; **NE**: nares to eye distance, from anterior-most point of eye to posterior-most point of nostril; **ED**: ear length, longest dimension of ear; **ForeaL**: forearm length, from base of palm to tip of elbow; **CrusL**: crus length, from base of heel to knee; **TrunkL**: trunk length, distance from axilla to groin measured from posterior edge of forelimb insertion to anterior edge of hindlimb insertion; **BW**: body width, the widest distance of body; **Internar**: internarial distance, distance between nares; **Interorb**: interorbital distance, shortest distance between left and right supraciliary scale rows.

Scale counts were taken as follows: **SL**: supralabials, counted from the first labial scale to corner of mouth; **IL**: infralabials, counted from the first labial scale to corner of mouth; **N**: nasal scales surrounding nare; **IN**: postrostrals or internasals; **PM**: postmentals; **GST**: granular scales surrounding dorsal tubercles; **V**: ventral scales in longitudinal rows at midbody; **SLB**: number of scales along the midbody from mental to anterior edge of cloaca; **FP**: femoral pores; **PP**: precloacal pores; **PAT**: postcloacal tubercles; **TubR**: tubercle, number of dorsal longitudinal rows of tubercles at midbody between the lateral folds; **EFS**: enlarged femoral scales, number of enlarged femoral scale beneath each thigh; **NSF IV**: number of subdigital lamellae on the fourth finger; **NST IV**: number of subdigital lamellae on the fourth toe. Bilateral scale counts were given as left/right; above sea level (asl).

### ﻿Multiple Factor Analysis (MFA)

The MFA was also applied in this study using morphometric and meristic characteristics, including SVL, HL, HW, HH, OrbD, SE, EE, ED, ForeaL, CrusL, TrunkL, Internar, Interob and SL, IL, GST, V, TubR, EFS, FP, PP, PAT, NSF IV, NST IV. Other morphological characteristics were not used due to the limitation of available morphometric and meristic data or incomplete sampling (regenerated tail). All statistical analyses were performed using R Core Team (2023). The MFA used six quantitative groups – “SVL”, “Head” (including HL, HW, HH), “Eye” (consist of OrbD, SE, EE, ED), “FT” (including ForeaL and CrusL), “TrunkL”, “Inter” (consist of Internar and Interorb) and eight qualitative groups – “SpeciesInfor” (including Name of species and ID), “SL-IL” (consist of SL and IL in both sides), “GST_PAT_TubR” (including GST, PAT in both sides and TubR), “V”, “EFS” in both sides, “FP” in both sides, “PP”, “LIV” (consist of NSF IV and NST IV in left side). To remove the effects of allometry, morphometric data were also normalized to adjust raw data of morphometrics through the allom() function in R package GroupStruct (available at heep://github.com/chankinonn/GroupStruct). Accordingly, the allometric formula is X_adj_ = log_10_(X) – ß[log_10_(SVL)-log_10_(SVL_mean_)], where X_adj_ = adjusted value; X = measured value; ß = unstandardized regression coefficient for each population and SVL_mean_ = overall average SVL of two populations (Thorpe 1975, 1983; Turan 1999; Lleonart et al. 2000; Grismer et al. 2021a; Chan and Grismer 2022). The ordination test was performed using packages Factoextra (Kassambara and Mundt 2017) and FactoMineR (Le et al. 2008) in the software R. The approach was applied to identify active groups and to explain phenotypic variance by estimating the first two Dim values-eigenvalue proportions. Similar coded colors in the MFA scatter plot, surrounded with convex hulls, were presented to visualize the phenotypic spaces of the new species and the most closely related species from China, namely *Cyrtodactylusgulinqingensis* Liu, Li, Hou, Orlov & Ananjeva, 2021; spaces were shown within a spatial coordinate of dimension axes (Dim1 and Dim2). To evaluate the overlap, the loadings of Dim1 and Dim2 of each *Cyrtodactylus* individual were extracted to identify the difference between the two species using the T-test. For all the tests, we applied a significance level of p < 0.05.

## ﻿Results

### ﻿Phylogenetic analysis

**Figure 2.**
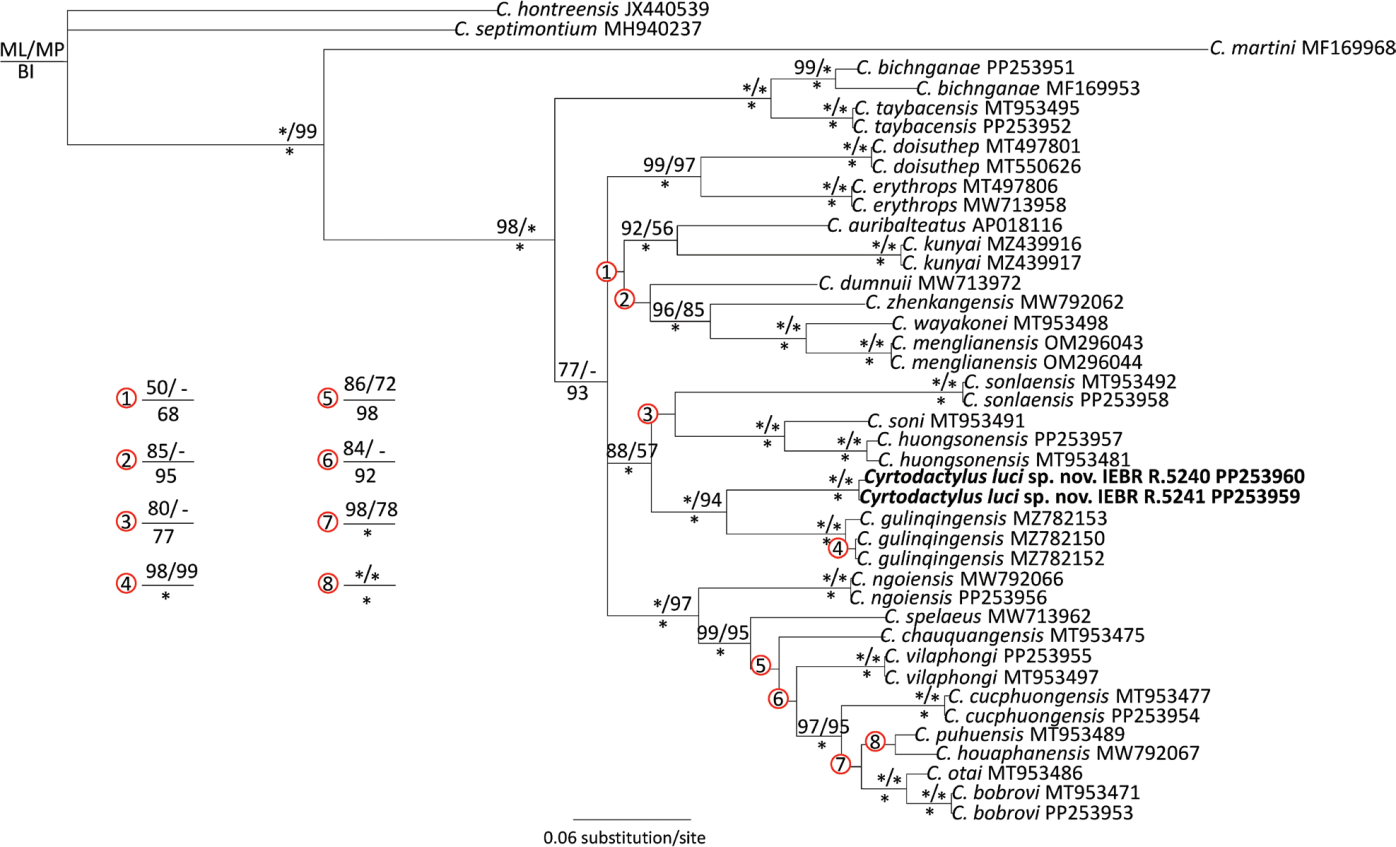
Phylogram based on the Bayesian analysis. Number above and below branches are ML/MP bootstrap and ultrafast bootstrap values and Bayesian posterior probabilities (≥ 50%), respectively. Asterisk and hyphen denote 100% and > 50% values, respectively.

The matrix of molecular data contained 1300 aligned characters, of which 580 were parsimony informative. The MP analysis produced a single most parsimonious tree (tree length = 2359, consistency index = 0.49, retention index = 0.66). Tree topologies from three analyses, ML, MP, and BI were similar and the *Cyrtodactylus* from Bac Ha District, Lao Cai Province was recovered with strong statistical support in all analyses as the sister taxon to *C.gulinqingensis* (BP = 94%; UBP = 100%; PP = 1.00) (Fig. 2). In terms of genetic divergences, the new species is separated from *C.gulinqingensis* by 8.87–9.22% based on a fragment of the mitochondrial ND2 gene. Genetically, it is also significantly divergent from other species within the *C.chauquangensis* group with a pairwise divergence of 12.32–23.85% (Suppl. material 1).

### ﻿Morphological analysis

**Figure 3.**
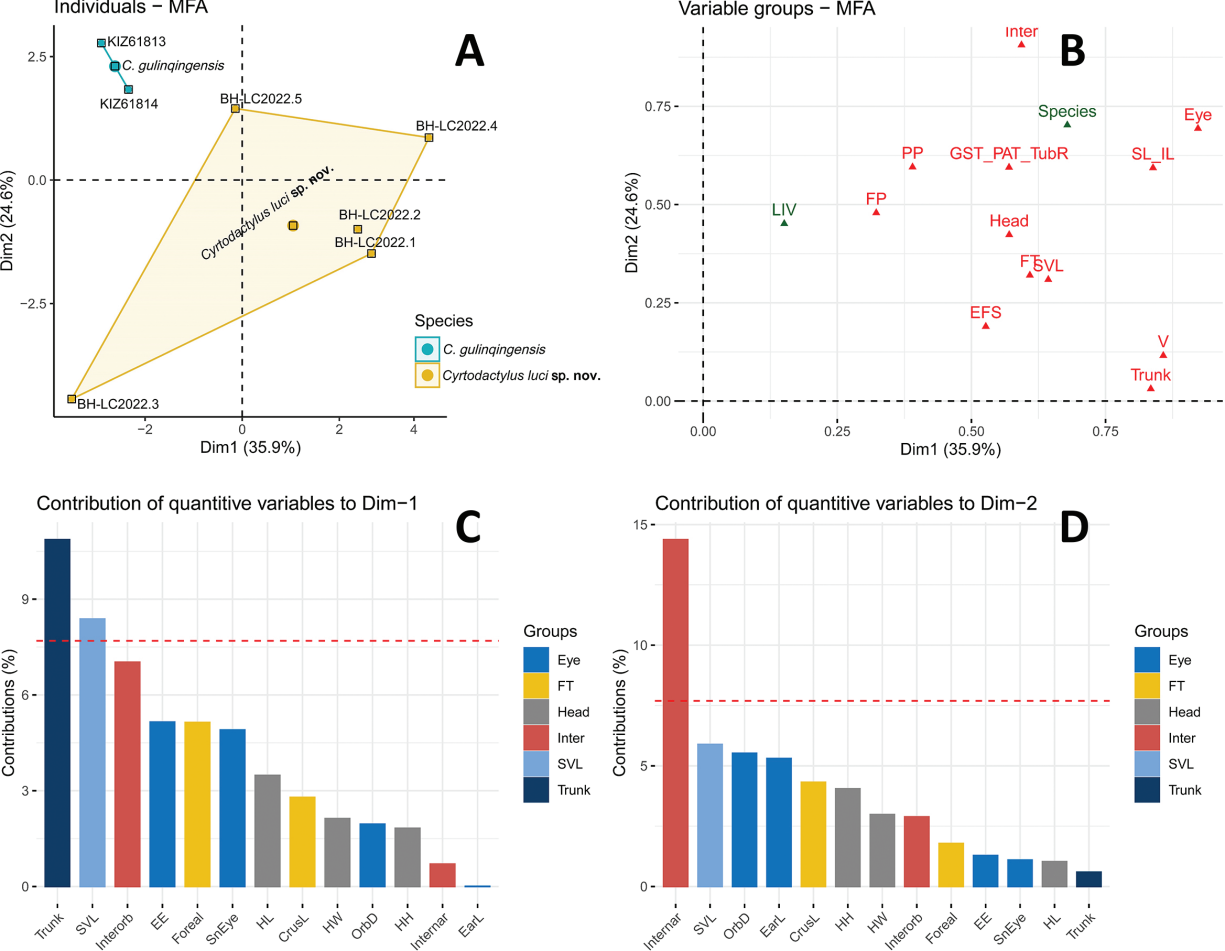
**A**MFA of *Cyrtodactylusluci* sp. nov. from Vietnam and *C.gulinqingensis* from China **B** scatterplot the groups of all variables for Dim1 and Dim2 axes in the MFA, green triangles as inactive groups of variables, red triangles as active groups of variables **C** bar plot of groups’ contribution to the first axes (Dim1) in the MFA**D** bar plot of groups’ contribution to the second axes (Dim2) in the MFA.

Morphologically, the new species from Bac Ha District, Lao Cai Province is closely similar to *C.gulinqingensis* from Yunnan Province, China, however, they plotted separately from each other in MFA (Fig. 3A) and there was a significant difference between two species (p < 0.05). The MFA also identified the data set of SVL, Head, Eye, FT, TrunkL, Inter, SL-IL, GST_PAT_TubR, V, EFS, FP, PP as active groups (Fig. 3B). The Eye, FT, Head, Inter, SVL and Trunk groups were the most important in both the first and second multi-factorial dimensions (Fig. 3C, D).

### ﻿Taxonomy

#### ﻿ Cyrtodactylus luci sp. nov.

Taxon classificationAnimaliaSquamataGekkonidae

B11D2BA8-985A-5319-B13B-49B4C453786E

https://zoobank.org/B03559F4-9C45-4991-8A74-5C346FCD6C37

Figs 4 , 5

##### Type material.

***Holotype*.**IEBR R.5237 (Field number BH-LC 2022.5), adult male, collected by T.T. Tran, T.Q. Phan and N.H. Nguyen on 30 June 2022, in limestone karst forest near Tham Phuc Village (22°29.514'N, 104°12.416'E, at an elevation of 677 m a.s.l), Coc Ly Commune, Bac Ha District, Lao Cai Province, Vietnam. ***Paratypes*.**IEBR R.5238 (Field number BH-LC 2022.1), IEBR R.5239 (Field number BH-LC 2022.3), adult males and IEBR R.5240, R.5241 (Field numbers BH-LC 2022.2, 2022.4), adult females, bear the same collection data as the holotype.

##### Diagnosis.

The new species can be distinguished from other members of the genus *Cyrtodactylus* by a combination of the following characteristics: Size medium (SVL up to 89.5 mm); dorsal tubercles in 17–19 irregular transverse rows; ventral scales in 32–34 longitudinal rows at midbody; precloacal pores present in both sexual, 9 or 10 in males, 8 or 9 in females; 12–15 enlarged femoral scales on each thigh; femoral pores 9–12 in males, 5–10 in females; postcloacal tubercles 2–4; lamellae under toe IV 21–23; dorsal pattern consisting of 5 or 6 irregular dark bands, a discontinuous thin neckband without V-shape or triangle shape in the middle, dorsal head surface with dark brown blotches; subcaudal scales transversely enlarged.

##### Description of holotype.

Adult male, snout-vent length (SVL) 86.3 mm; body relatively short (TrunkL/SVL 0.4); head distinct from neck, moderately long (HL/SVL 0.28), relatively wide (HW/HL 0.69), slightly depressed (HH/HL 0.41); eye slightly large (OrbD/HL 0.24), pupils vertical; upper eyelid fringe with spinous scales; ear opening below the postocular stripes, obliquely directed and oval, small in size (ED/HL 0.06); two enlarged supranasals, separated from each other anteriorly by one internasal; nares oval, surrounded by supranasal, rostral, first supralabial and three postnasals; loreal region and frontal concave; snout long (SE/HL 0.41), round anteriorly, longer than diameter of orbit (OrbD/SE 0.58); snout scales small, round, granular, larger than those in frontal and parietal regions; rostral wider than high with a medial suture, bordered by first supralabial on each side, nostrils, two supranasals and one internasal; mental triangular, wider than high; postmentals two, enlarged, in contact posteriorly, bordered by mental anteriorly, first infralabial laterally, and an enlarged chin scale posteriorly; supralabials 11/10; infralabials 11/10.

Dorsal scales granular; dorsal tubercles round, keeled, conical, four or five times larger than the size of adjoining scales, each surrounded by 10 granular scales, tubercles forming 17 irregular longitudinal rows at midbody; ventral scales smooth, medial scales 2–3 times larger than dorsal granules, round, subimbricate, largest posteriorly, in 32 longitudinal rows at midbody; lateral folds present, without interspersed tubercles; gular region with homogeneous smooth scales; ventral scales between mental and cloacal slit 170; precloacal groove absent; three rows of enlarged scales present in posterior region of pore-bearing scales; ten precloacal pores arranged in a chevron; 12 or 13 enlarged femoral scales beneath thighs continuous with pore-bearing precloacal scales; femoral pores present on each enlarged femoral scales (except one on right thigh), 24 in total; precloacal pores large, horizontal elongated, positioned in posterior margin of scales; femoral pores small, round, positioned in the center of scales.

Fore and hind limbs moderately slender (ForeaL/SVL 0.16, CrusL/SVL 0.19); dorsal surface of forelimbs covered by few slightly developed tubercles; fingers and toes lacking distinct webbing; subdigital lamellae: finger I 12, finger II 16, finger III 17, finger IV 20, finger V 18, toe I 12, toe II 17, toe III 20, toe IV 21, toe V 20.

Tail regenerated, 104.5 mm in length (generated part 19.5 mm); longer than snout-vent length (TaL/SVL: 1.21); postcloacal tubercles 4/4; subcaudals on original part of tail distinctly transversely enlarged, flat, smooth.

Coloration in life. Ground color of dorsal surface of head, neck, body, limbs and tail light brown. Dorsal surface of head with some dark brown blotches; labial region brown with yellowish cream stripes; skin above the eye gray; eyelid with light yellow color; iris yellow copper with black marking; pupil vertical, elliptical, black; nuchal loop dark brown, discontinous, extending from posterior corner of eye to the neck; tubercles on head, limbs, dorsum light brown to yellow; dorsum with five irregularly-shaped transversal bands and additional irregular smaller blotches; upper surface of limbs with irregular brown marks; six dark brown irregular bands on original part of tail while regenerated part of tail dark gray; chin, throat, chest, belly, lower limbs and ventral surface of tail cream.

Coloration in preservative. The overall color scheme slightly fades in 70% alcohol; yellow color disappeared in preservation while main characteristics are still clearly discernible; dorsal ground color of head, neck, body, limbs and tail grayish brown; color of chin, throat, chest, belly and lower limbs did not change noticeably in preservation.

##### Sexual dimorphism and variation.

**Figure 4.**
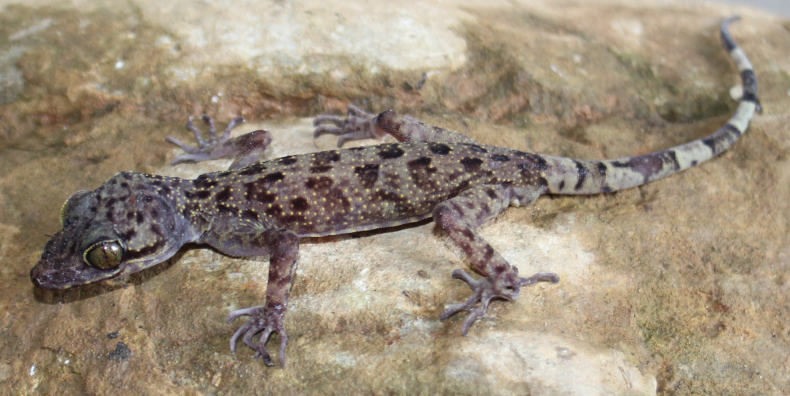
Male holotype of *Cyrtodactylusluci* sp. nov. (IEBR R.5237) in life. Photo: T.Q. Phan.

**Figure 5.**
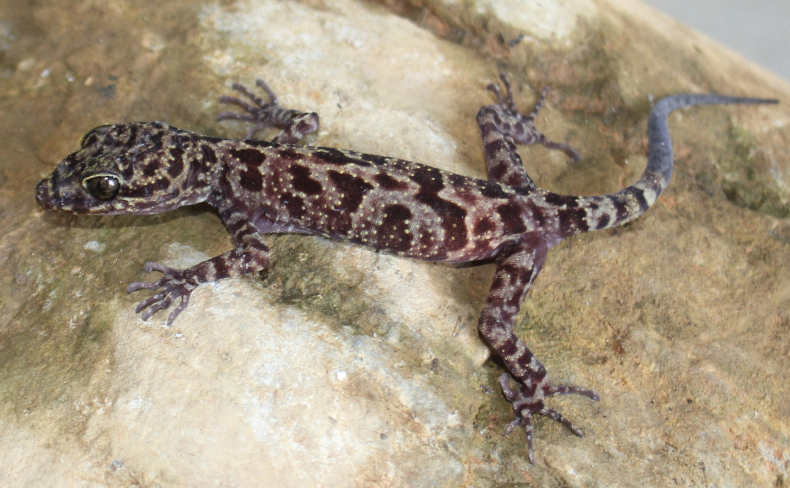
Female paratype of *Cyrtodactylusluci* sp. nov. (IEBR R.5241) in life. Photo: T.Q. Phan.

The males differ from females in the shape of precloacal pores (larger in males), and the presence of hemipenial swellings at the tail base. For other morphological characteristics see Table 2, Figs 4, 5.

**Table 2.** Measurements (in mm) and morphological characteristics (abbreviations as in Material and methods) of the type series of *Cyrtodactylusluci* sp. nov. (* = regenerated or broken tail); bilateral meristic characteristics are given as (left/right).

Characters	IEBR R.5237	IEBR R.5238	IEBR R.5239	IEBR R.5240	IEBR R.5241	Min–Max
	(Holotype)	(Paratype)	(Paratype)	(Paratype)	(Paratype)	
Sex	M	M	M	F	F	
SVL	86.3	88.7	71.7	87.1	89.5	71.7–89.5
TaL	104.5*	107.7	86.2	84.2*	84.1*	86.2–107.7
HL	24.5	24.0	20.3	24.6	25.2	20.3–25.2
HW	16.9	16.6	12.8	17.4	17.4	12.8–17.4
HH	10.1	9.8	7.1	9.7	10.6	7.1–10.6
OrbD	5.9	4.9	4.7	5.1	4.8	4.7–5.9
SE	10.2	10.0	8.4	10.6	10.8	8.4–10.8
EE	6.5	6.6	5.5	6.6	7.2	5.5–7.2
NE	7.5	7.9	6.0	7.7	8.7	6.0–8.7
ED	1.4	1.6	1.9	1.8	1.3	1.4–1.9
ForeaL	14.2	14.2	11.5	14.1	14.4	11.5–14.4
CrusL	16.3	17.2	13.5	16.7	16.8	13.5–17.2
TrunkL	34.4	39.7	31.5	39.7	42.1	31.5–42.1
BW	13.8	14.0	9.4	17.6	19.2	9.4–19.2
Internar	2.8	2.5	2.0	2.7	3.0	2.0–3.0
Interorb	6.9	7.3	5.2	7.6	7.8	5.2–7.8
SL	11/10	11/11	10//10	11/10	11/9	9–11
IL	11/10	12/12	11/13	11/10	9/12	9–13
N	4/4	4/4	4/4	4/4	4/5	4–5
IN	1	1	1	1	1	1
PM	2	3	2	2	2	2
GST	10/10/10	10/10/10	10/9/10	10/10/10	10/10/10	9–10
V	32	34	32	34	34	32–34
SLB	170	171	169	171	166	166–171
FP	12/12	10/9	11/12	10/10	7/5	9–12 in males 5–10 in females
PP	10	9	9	8	9	9–10 in males 8–9 in females
PAT	3/3	4/2	3/3	4/3	3/3	2–4
TubR	17	17	17	19	18	17–19
EFS	13/12	14/15	14/14	13/13	17/15	12–15
NSF IV	18	21	20	19	20	18–21
NST IV	21	23	23	21	23	21–23

##### Distribution.

*Cyrtodactylusluci* sp. nov. is currently known only from the type locality in Bac Ha District, Lao Cai Province, Vietnam (Fig. 1).

##### Etymology.

The species was named after the zoologist from the Vietnam National Museum of Nature, Vietnam Academy of Science and Technology, late Associate Professor Doctor Luc Van Pham, who contributed greatly to the biodiversity study in Vietnam. For the common names, we suggest Luc’s Bent-toed Gecko (English) and Thạch sùng ngón lực (Vietnamese).

##### Natural history.

**Figure 6.**
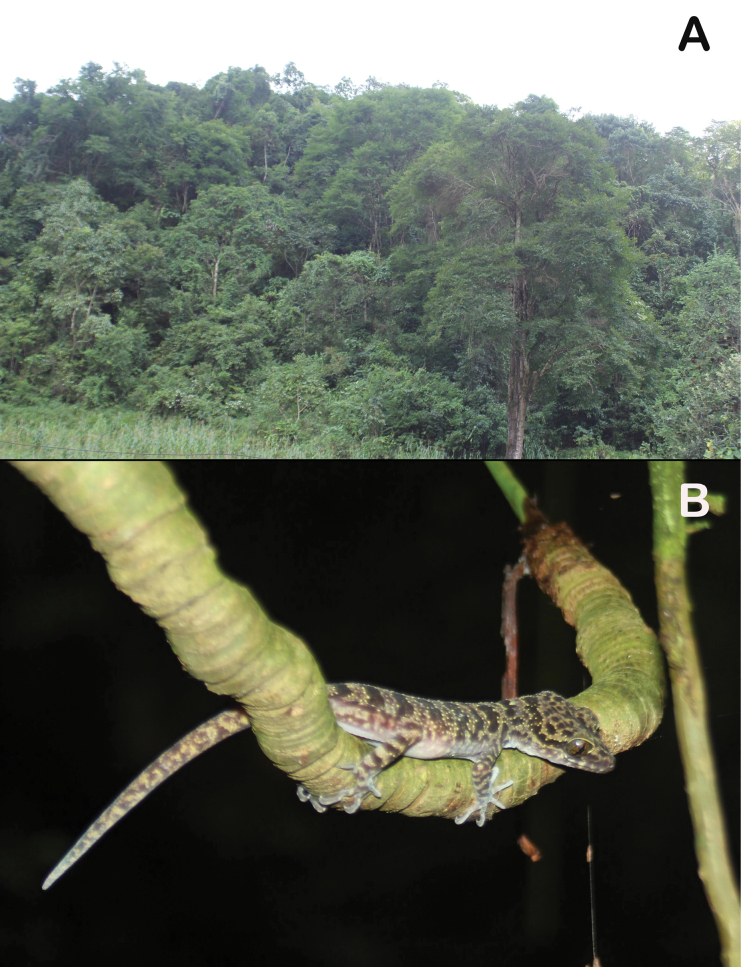
**A** macrohabitat **B** microhabitat of *Cyrtodactylusluci* sp. nov. Coc Ly Commune, Bac Ha District, Lao Cai Province, Vietnam. Photo: T.Q. Phan.

The bent-toed geckos were collected between 19:00 and 22:00, both on limestone cliffs and on trees, about 1.0–1.8 m above the ground. The surrounding habitat was secondary karst forest of medium and small hardwoods mixed with shrubs and vines (Fig. 6). Air temperature was 25.9 °C and relative humidity was 92%.

##### Comparisons.

*Cyrtodactylusluci* sp. nov. is distinguishable from all other members of the *C.chauquangensis* species group by a unique combination of morphological characteristics.

*Cyrtodactylusluci* sp. nov. differs from *C.auribalteatus* Sumontha, Panitvong & Deein, 2010 by having fewer ventral scale rows (32–34 vs. 38–40 in *C.auribalteatus*), more enlarged femoral scales on each side (12–15 vs. 5–7 in *C.auribalteatus*), more femoral pores on each side in males (9–12 vs. 4 or 5 in *C.auribalteatus*), the presence of femoral pores on each side in females (5–10 vs. absent in *C.auribalteatus*), more precloacal pores in males (9 or 10 vs. 6 in *C.auribalteatus*), the presence of precloacal pores in females (8 or 9 vs. absent in *C.auribalteatus*) and fewer dorsal tubercle rows (17–19 vs. 22–24 in *C.auribalteatus*); from *C.bichnganae* Ngo & Grismer, 2010 by having a smaller size (SVL 71.7–89.5 mm vs. 95.3–99.9 mm in *C.bichnganae*), more ventral scale rows (32–34 vs. 30 or 31 in *C.bichnganae*), more femoral pores on each side in females (5–10 vs. 1 in *C.bichnganae*), and more lamellae under toe IV (21–23 vs. 16–20 in *C.bichnganae*); from *C.bobrovi* Nguyen, Le, Pham, Ngo, Hoang, Pham & Ziegler, 2015 by having fewer ventral scale rows (32–34 vs. 40–45 in *C.bobrovi*), the presence of enlarged femoral scales on each side (12–15 vs. absent in *C.bobrovi*), the presence of femoral pores on each side in males (9–12 vs. absent in *C.bobrovi*) and in females (5–10 vs. absent in *C.bobrovi*), more precloacal pores in males (9 or 10 vs. 5 in *C.bobrovi*), the presence of precloacal pores in females (8 or 9 vs. absent in *C.bobrovi*), and the presence of transversely enlarged subcaudal plates (vs. absent in *C.bobrovi*); from *C.chauquangensis* Hoang, Orlov, Ananjeva, Johns, Hoang & Dau, 2007 by having a smaller size (SVL 71.7–89.5 mm vs. 91.0–99.3 mm in *C.chauquangensis*), fewer ventral scale rows (32–34 vs. 36–38 in *C.chauquangensis*), the presence of enlarged femoral scales on each side (12–15 vs. absent in *C.chauquangensis*), the presence of femoral pores on each side in males (9–12 vs. absent in *C.chauquangensis*) and also in females (5–10 vs. absent in *C.chauquangensis*), more precloacal pores in males (9 or 10 vs. 6 or 7 in *C.chauquangensis*) and also in females (8 or 9 vs. 6 or 7 in *C.chauquangensis*); from *C.cucphuongensis* Ngo & Chan, 2011 by having fewer ventral scale rows (32–34 vs. 42 in *C.cucphuongensis*), the presence of femoral pores on each side in males (9–12 vs. absent in *C.cucphuongensis*) and in females (5–10 vs. absent in *C.cucphuongensis*) and the presence of precloacal pores in males (9–10 vs. absent in *C.cucphuongensis*); from *C.doisuthep* Kunya, Panmongkol, Pauwels, Sumontha, Meewasana, Bunkhwamdi & Dangsri, 2015 by the presence of femoral pores on each side in males (9–12 vs. absent in *C.doisuthep*) and in females (5–10 vs. absent in *C.doisuthep*), more precloacal pores in males (9 or 10 vs. 5 or 6 in *C.doisuthep*) and also in females (8 or 9 vs. absent in *C.doisuthep*); from *C.dumnuii* Bauer, Kunya, Sumontha, Niyomwan, Pauwels, Chanhome & Kunya, 2010 by having fewer ventral scale rows (32–34 vs. 40 in *C.dumnuii*), more femoral pores on each side in males (9–12 vs. 6–7 in *C.dumnuii*) and in females (5–10 vs. absent in *C.dumnuii*), more precloacal pores in males (9 or 10 vs. 5 or 6 in *C.dumnuii*) and also in females (8 or 9 vs. 0–7 in *C.dumnuii*) and more lamellae under toe IV (21–23 vs. 19 in *C.dumnuii*); from *C.erythrops* Bauer, Kunya, Sumontha, Niyomwan, Panitvong, Pauwels, Chanhome & Kunya, 2009 by having more ventral scale rows (32–34 vs. 28 in *C.erythrops*), more lamellae under finger IV (18–21 vs. 16 in *C.erythrops*), more lamellae under toe IV (21–23 vs. 20 in *C.erythrops*) and differences in dorsal color pattern (banded vs. blotched in *C.erythrops*); from *C.gulinqingensis* Liu, Li, Hou, Orlov & Ananjeva, 2021 by having more dorsal tubercle rows (17–19 vs. 14–16 in *C.gulinqingensis*), fewer femoral pores on each side in males (9–12 vs. 13–15 in *C.gulinqingensis*) and in females (5–10 vs. 1–3 in *C.gulinqingensis*) and fewer precloacal pores in females (8 or 9 vs. 7 in *C.gulinqingensis*); from *C.houaphanensis* Schneider, Luu, Sitthivong, Teynié, Le, Nguyen & Ziegler, 2020 by having fewer ventral scale rows (32–34 vs. 35 in *C.houaphanensis*), the presence of enlarged femoral scales on each side (12–15 vs. absent in *C.houaphanensis*), the presence of femoral pores on each side in males (9–12 vs. absent in *C.houaphanensis*) and in females (5–10 vs. absent in *C.houaphanensis*) and more precloacal pores in males (9 or 10 vs. 6 in *C.houaphanensis*); from *C.huongsonensis* Luu, Nguyen, Do & Ziegler, 2011 by having fewer ventral scale rows (32–34 vs. 41–48 in *C.huongsonensis*), more enlarged femoral scales on each side (12–15 vs. 7–9 in *C.huongsonensis*) and more precloacal pores in males (9 or 10 vs. 6 in *C.huongsonensis*); from *C.martini* Ngo, 2011 by having fewer ventral scale rows (32–34 vs. 39–43 in *C.martini*), more precloacal pores in males (9 or 10 vs. 4 in *C.martini*), the presence of precloacal pores in females (8 or 9 vs. absent in *C.martini*) and the presence of transversely enlarged subcaudal plates (vs. absent in *C.martini*); from *C.menglianensis* Liu & Rao, 2022 by having more ventral scale rows (32–34 vs. 26–29 in *C.menglianensis*), the presence of enlarged femoral scales on each side (12–15 vs. absent in *C.menglianensis*), the presence of femoral pores on each side in males (9–12 vs. absent in *C.menglianensis*) and in females (5–10 vs. absent in *C.menglianensis*), more precloacal pores in males (9 or 10 vs. 7 in *C.menglianensis*) and the presence of precloacal pores in females (8 or 9 vs. absent in *C.menglianensis*); from *C.ngoiensis* Schneider, Luu, Sitthivong, Teynié, Le, Nguyen & Ziegler, 2020 by having fewer ventral scale rows (32–34 vs. 38–43 in *C.ngoiensis*), more enlarged femoral scales on each side (12–15 vs. 7–10 in *C.ngoiensis*), more femoral pores on each side in males (9–12 vs. 7 in *C.ngoiensis*) and in females (5–10 vs. absent in *C.ngoiensis*), more precloacal pores in males (9 or 10 vs. 7 in *C.ngoiensis*) and in females (8 or 9 vs. 7 in *C.ngoiensis*) and more lamellae under toe IV (21–23 vs. 19–20 in *C.ngoiensis*); from *C.otai* Nguyen, Le, Pham, Ngo, Hoang, Pham & Ziegler, 2015 by having fewer ventral scale rows (32–34 vs. 38–43 in *C.otai*), the presence of enlarged femoral scales on each side (12–15 vs. absent in *C.otai*), the presence of femoral pores on each side in males (9–12 vs. absent in *C.otai*) and in females (5–10 vs. absent in *C.otai*), more precloacal pores in males (9 or 10 vs. 7 or 8 in *C.otai*), the presence of precloacal pores in females (8 or 9 vs. absent in *C.otai*), and the presence of transversely enlarged subcaudal plates (vs. absent in *C.otai*); from *C.puhuensis* Nguyen, Yang, Le, Nguyen, Orlov, Hoang, Nguyen, Jin, Rao, Hoang, Che, Murphy & Zhang, 2014 by having fewer ventral scale rows (32–34 vs. 36 in *C.puhuensis*), the presence of femoral pores on each side in males (9–12 vs. absent in *C.puhuensis*) and in females (5–10 vs. absent in *C.puhuensis*), and more precloacal pores in males (9 or 10 vs. 5 in *C.puhuensis*); from *C.soni* Le, Nguyen, Le & Ziegler, 2016 by having fewer ventral scale rows (32–34 vs. 41–45 in *C.soni*), more dorsal tubercle rows (17–19 vs. 10–13 in *C.soni*), more enlarged femoral scales on each side (12–15 vs. 8–11 in *C.soni*), more femoral pores on each side in males (9–12 vs. 6–8 in *C.soni*), and more precloacal pores in males (9 or 10 vs. 6 or 7 in *C.soni*); from *C.sonlaensis* Nguyen, Pham, Ziegler, Ngo & Le, 2017 by having more dorsal tubercle rows (17–19 vs. 13–15 in *C.sonlaensis*), fewer femoral pores on each side in males (9–12 vs. 14–15 in *C.sonlaensis*), the presence of femoral pores on each side in females (5–10 vs. absent in *C.sonlaensis*), more precloacal pores in males (9 or 10 vs. 8 in *C.sonlaensis*) and the presence of precloacal pores in females (8 or 9 vs. absent in *C.sonlaensis*); from *C.spelaeus* Nazarov, Poyakov, Orlov, Nguyen, Milto, Martynov, Konstantinov & Chulisov, 2014 by having fewer ventral scale rows (32–34 vs. 36–39 in *C.spelaeus*), the presence of enlarged femoral scales on each side (12–15 vs. absent in *C.spelaeus*), the presence of femoral pores on each side in males (9–12 vs. absent in *C.spelaeus*) and in females (5–10 vs. absent in *C.spelaeus*) and differences in dorsal color pattern (banded vs. blotched in *C.spelaeus*); from *C.taybacensis* Pham, Le, Ngo, Ziegler & Nguyen, 2019 by having more dorsal tubercle rows (17–19 vs. 13–16 in *C.taybacensis*), the presence of femoral pores on each side in males (9–12 vs. absent in *C.taybacensis*) and in females (5–10 vs. absent in *C.taybacensis*), fewer precloacal pores in males (9 or 10 vs. 11–13 in *C.taybacensis*) and more lamellae under toe IV (21–23 vs. 16–20 in *C.taybacensis*); from *C.vilaphongi* Schneider, Nguyen, Le, Nophaseud, Bonkowski & Ziegler, 2014 by having more dorsal tubercle rows (17–19 vs. 15–16 in *C.vilaphongi*), the presence of enlarged femoral scales on each side (12–15 vs. absent in *C.vilaphongi*), the presence of femoral pores on each side in females (5–10 vs. absent in *C.vilaphongi*) and in females (8 or 9 vs. absent in *C.vilaphongi*), more lamellae under toe IV (21–23 vs. 18–20 in *C.vilaphongi*), and the presence of transversely enlarged subcaudal plates (vs. absent in *C.vilaphongi*); from *C.wayakonei* Nguyen, Kingsada, Rosler, Auer & Ziegler, 2010 by the presence of enlarged femoral scales on each side (12–15 vs. absent in *C.wayakonei*), the presence of femoral pores on each side in males (9–12 vs. absent in *C.wayakonei*) and in females (5–10 vs. absent in *C.wayakonei*), more precloacal pores in males (9 or 10 vs. 6–8 in *C.wayakonei*) and in females (8 or 9 vs. 7 in *C.wayakonei*), and more lamellae under toe IV (21–23 vs. 19–20 in *C.wayakonei*); from *C.zhenkangensis* Liu & Rao, 2021 by having fewer dorsal tubercle rows (17–19 vs. 20–24 in *C.zhenkangensis*), more femoral pores on each side in males (9–12 vs. 2–5 in *C.zhenkangensis*) and in females (5–10 vs. 0–3 in *C.zhenkangensis*) and the presence of dark-colored nuchal loop (vs. absent in *C.zhenkangensis*).

## ﻿Discussion

The new species from Bac Ha District, Lao Cai Province, is most similar to *Cyrtodactylusgulinqingensis*, a recently described species from Muguan County, Wenshan Prefecture, Yunnan Province of China (Liu et al. 2021). In terms of geographic distribution, the type locality of *C.luci* is approximately 40 km distant from that of its sister species in China. However, they are distinguished from each other by morphological differences as well as a genetic divergence of 8.87–9.22% (ND2 gene).

Our tree topology (Fig. 2) is similar to that reported in Grismer et al. (2021b). However, while *C.auribalteatus* is recovered as a member of the clade including *C.dumnuii*, *C.wayakonei* and other taxa in this study, it is grouped with the lineage consisting of *C.sonlaensis*, *C.huongsonensis* and *C.soni* in Grismer et al. (2021b). According to our phylogenetic analyses, the new species and *C.gulinqingensis* from Yunnan cluster with the latter clade with strong nodal support provided only by BI (Fig. 2). In addition to *C.luci* and *C.gulinqingensis*, the other species in the group occur in Son La (*C.sonlaensis*) and Ninh Binh (*C.soni*) provinces and the suburb of Ha Noi City (*C.huongsonensis*), northwestern Vietnam.

In the *Cyrtodactyluschauquangensis* group, except for *C.doisuthep*, a species known from dry evergreen and deciduous dipterocarp forests in Thailand (Kunya et al. 2014), all 23 remaining species are karst dwellers, comprising three species from Yunnan Province of China, five species from northern Laos, four species from northern Thailand, and 12 species from northern Vietnam (Uetz et al. 2023, this study). In terms of altitudinal distribution range, the members of this species group are found at elevations from 17 m (*C.soni*) to 1660 m (*C.doisuthep*) but most of them occur at elevations between 300 and 800 m a.s.l (Kunya et al. 2015; Le et al. 2016). The new species is the 24^th^ species of the *C.chauquangensis* group, the first species from Lao Cai Province and the eastern side of the Red River in Vietnam, and the 53^rd^ species of *Cyrtodactylus* known from Vietnam (Ngo et al. 2022; Uetz et al. 2023).

## Supplementary Material

XML Treatment for Cyrtodactylus luci

## ﻿Additional information

### Author ORCIDs

Tung Thanh Tran https://orcid.org/0000-0001-7648-1179

Quyen Hanh Do https://orcid.org/0000-0002-9437-4673

Cuong The Pham https://orcid.org/0000-0001-5158-4526

Tien Quang Phan https://orcid.org/0000-0002-2738-5364

Hanh Thi Ngo https://orcid.org/0000-0002-5283-6243

Minh Duc Le https://orcid.org/0000-0002-2953-2815

Thomas Ziegler https://orcid.org/0000-0002-4797-609X

Truong Quang Nguyen https://orcid.org/0000-0002-6601-0880

### Data availability

All of the data that support the findings of this study are available in the main text or Supplementary Information.

## Supplementary materials

Supplementary material 1 Pair-wise genetic distance between samples used in this study Data type xlsx https://binary.pensoft.net/file/986588This dataset is made available under the Open Database License (http://opendatacommons.org/licenses/odbl/1.0/). The Open Database License (ODbL) is a license agreement intended to allow users to freely share, modify, and use this Dataset while maintaining this same freedom for others, provided that the original source and author(s) are credited.Tung Thanh Tran, Quyen Hanh Do, Cuong The Pham, Tien Quang Phan, Hanh Thi Ngo, Minh Duc Le, Thomas Ziegler, Truong Quang Nguyen
